# A mathematical model for dynamics of soluble form of DNAM-1 as a biomarker for graft-versus-host disease

**DOI:** 10.1371/journal.pone.0228508

**Published:** 2020-02-10

**Authors:** Yuki Goshima, Shinji Nakaoka, Kazuteru Ohashi, Hisashi Sakamaki, Kazuko Shibuya, Akira Shibuya

**Affiliations:** 1 Department of Immunology, Faculty of Medicine, University of Tsukuba, Tsukuba, Japan; 2 School of Medicine, University of Tsukuba, Tsukuba, Japan; 3 Faculty of Advanced Life Science, Hokkaido University, Sapporo, Hokkaido, Japan; 4 PRESTO, Japan Science and Technology Agency, Saitama, Japan; 5 Hematology Division, Tokyo Metropolitan Cancer and Infectious Diseases Center Komagome Hospital, Tokyo, Japan; 6 R&D Center for Innovative Drug Discovery, University of Tsukuba, Tsukuba, Ibaraki, Japan; 7 Life Science Center for Survival Dynamics, Tsukuba Advanced Research Alliance (TARA), University of Tsukuba, Tsukuba, Japan; University of Kentucky, UNITED STATES

## Abstract

DNAM-1 (CD226) is an activating immunoreceptor expressed on T cells and NK cells and involved in the pathogenesis of acute graft-versus-host disease (aGVHD) after allogeneic hematopoietic stem cell transplantation (allo-HSCT). We previously reported that a soluble form of DNAM-1 (sDNAM-1) is generated by shedding from activated T cells. Moreover, higher serum levels of sDNAM-1 in patients before allo-HSCT is a predictive biomarker for the development of aGVHD based on the retrospective univariate and multivariate analyses in allo-HSCT patients. However, it remains unclear how the serum levels of sDNAM-1 are regulated after allo-HSCT and whether they are associated with the development of aGVHD. Here, we constructed a mathematical model to assess the dynamics of sDNAM-1 after allo-HSCT by assuming that there are three types of sDNAM-1 (the first and the second were from alloreactive and non-alloreactive donor lymphocytes, respectively, and the third from recipient lymphocytes). Our mathematical model fitted well to the data set of sDNAM-1 in patients (n = 67) who had undergone allo-HSCT and suggest that the high proportion of the first type of sDNAM-1 to the total of the first and second types is associated with high risk of the development of severe aGVHD. Thus, sDNAM-1 after allo-HSCT can be a biomarker for the development of aGVHD.

## Introduction

Acute graft-versus-host disease (aGVHD) is a major complication of allogeneic hematopoietic stem cell transplantation (allo-HSCT). Although the mechanism underlying the development of aGVHD has been extensively studied in vitro and in vivo [1–3], the diagnosis of and treatment for aGVHD are still problematic.

DNAM-1, also known as CD226, is an activating immunoreceptor expressed on CD4^+^ T cells, CD8^+^ T cells, natural killer (NK) cells, and monocytes [4]. We and others demonstrated that DNAM-1 plays an important role in the development of aGVHD in mouse models [5,6]. Moreover, we have recently identified a soluble form of DNAM-1 (sDNAM-1), which is shed from the membrane type of DNAM-1 expressed on the cell surface of activated T lymphocytes, in human sera [7]. We performed retrospective univariate and multivariate analyses of serum levels of sDNAM-1 in patients before and after allo-HSCT at a single center (n = 71) [7]. We demonstrated that cumulative incidences of all grade (grade I–IV) and grade II–IV aGVHD in patients with high maximal serum levels of sDNAM-1 (≥30 pM) in the 7 days before allo-HSCT were significantly higher than those in patients with low maximal serum levels of sDNAM-1 (<30 pM) in the same period, and concluded that the serum levels of sDNAM-1 can be a predictive biomarker for the development of aGVHD [7]. However, it remains unclear how the dynamics of serum levels of sDNAM-1 after allo-HSCT is regulated and whether it is associated with the development of aGVHD.

In this study, we constructed a mathematical model to assess the dynamics of serum levels of sDNAM-1, and revisited the data set of sDNAM-1, which had been analyzed previously [7], particularly after, rather than before, allo-HSCT to be applied by the mathematical model. We show that sDNAM-1 after allo-HSCT can be a biomarker for the development of aGVHD.

## Materials and methods

### Patients, samples, and inclusion criteria

**Fig 1:**
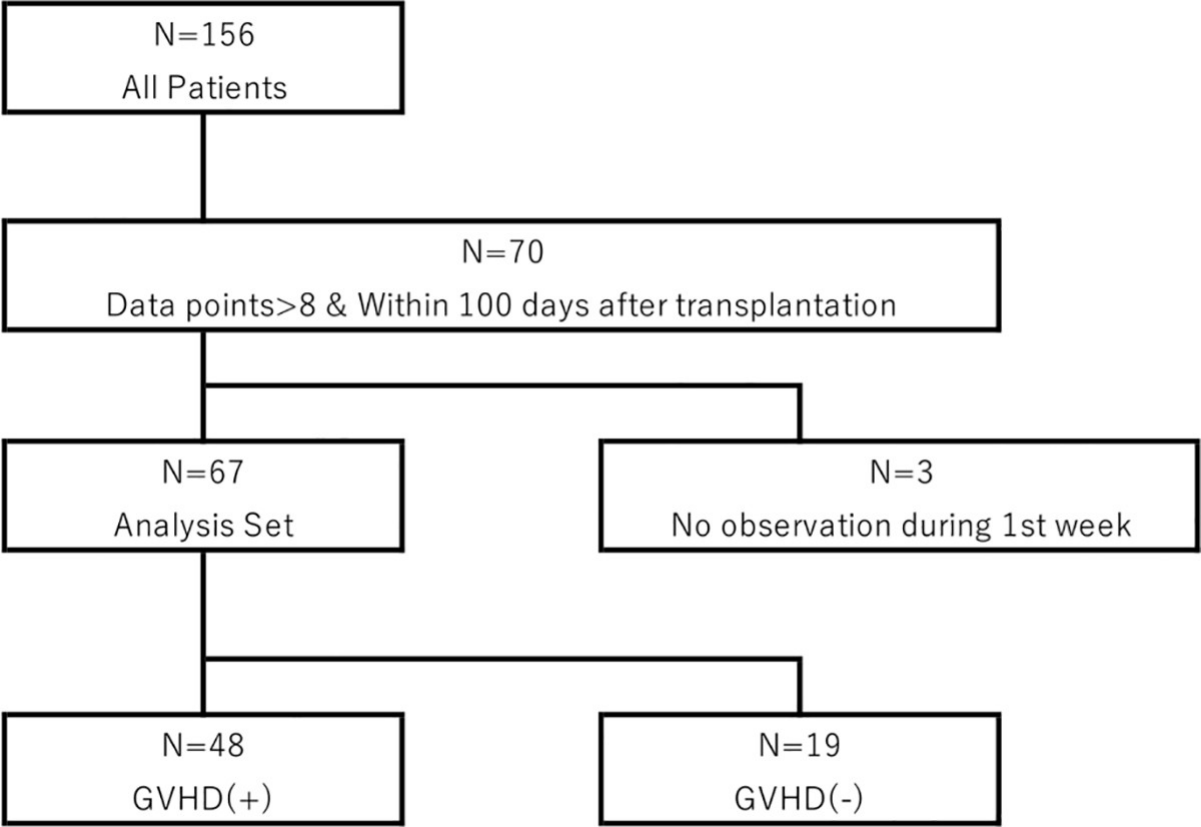
Patient disposition. From 156 patients analyzed for sDNAM-1 in the sera, we finally included 67 in our study. Details of the inclusion criteria are given in the Materials and Methods.

Serum samples were obtained from 156 patients at the Tokyo Metropolitan Cancer and Infectious Diseases Center, Komagome Hospital, Japan, between March 2009 and November 2011. Data from some of the patients had already been analyzed [7]; we basically followed their methods in terms of such features as informed consent and sample handling but here we included other patients according to our new criteria which is shown in **Fig 1**. Written informed consent was obtained from each patient in accordance with the Declaration of Helsinki. This study was approved by the ethics committee of the University of Tsukuba (approval No., 505) and Tokyo Metropolitan Cancer and Infectious Diseases Center, Komagome Hospital (approval No., 571). Soluble DNAM-1 in sera was measured by sandwich enzyme-linked immunosorbent assay (ELISA), as described [7]. The data were collected from about 7 days before allo-HSCT up to a maximum of 249 days after allo-HSCT. Since our mathematical model required eight parameters and at least one parameter within 100 days and 7 days after allo-HSCT, respectively, we excluded patients who did not meet these criteria. As a result, 67 patients (GVHD (+) = 48 patients, GVHD (-) = 19 patients) satisfied our criteria (**Fig 1**); their characteristics are listed in **Table 1**.

**Table 1:** Patient characteristics and clinical information.

Variables	GVHD(–) (n = 19)	GVHD(+) (n = 48)	*P*-value
Age	48.4 (± 13.9)	44.7 (± 13.8)	0.42
TBI(+)	9	30	0.26
TBI(–)	10	18	
BMT	16	37	0.74
Other	3	11	
RD	2	10	0.49
URD	17	38	
MA	12	33	0.77
RIC	7	15	
*Allele*			0.78
Full match	12	32	
Mismatch	7	16	
*Treatment*			0.58
Tacrolimus	11	32	
CsA	8	16	
AML	10	18	-
ALL	1	12	
MDS	3	6	
Others	5	12	
*GVHD* [8]			–
Grade 1	–	17	
Grade 2	–	22	
Grade 3	–	8	
Grade 4	–	1	

There were no significant differences in the listed variables between GVHD (–) and GVHD (+) patients. TBI; Total Body Irradiation, BMT; Bone Marrow Transplantation, RD; Related Donor, URD; Unrelated Donor, MA; Myeloablative, RIC; Reduced Intensity Conditioning, CsA; Cyclosporin A.

### Definition of grading system of acute GVHD and transplantation procedures

The GVHD grade of each patient was determined as previously described [8,9]. The transplantation procedures are described previously [7].

### Mathematical model and statistical analysis

sDNAM-1 was detected in the sera of healthy people as well as patients before and after allo-HSCT. sDNAM-1 might be produced by either donor cells, recipient cells or both. Therefore, we modeled the dynamics of those cells using ordinary differential equations. Differential equations have been used for describing the virus and cell growth [10,11]. In our research, we firstly formulate the mathematical model to explain the dynamics of sDNAM-1. Secondly, we fitted this model to the patients’ data and got each patient’s parameter using Package FME [12] in R (version 3.4.3) *(R Core Team (2018))*. *R*: *A language and environment for statistical computing*. *R Foundation for Statistical Computing*, *Vienna*, *Austria*. *URL https://www.R-project.org/)*. As for statistical analysis, a *t*-test, Fisher’s exact test and Binomial test were used to analyze the value of *R*_*day_n*_, patient demographic and clinical information, and comparison of Akaike’s Information Criterion (AIC) between two different mathematical models, respectively. For all tests, a *P*-value of less than 0.05 was considered as statistical significance.

#### Assessing goodness of fit to data and contribution of first type of sDNAM-1 to aGVHD

We compared 2 mathematical models to assess their goodness of fit to the data. The model 1 is consisted of two secretors of sDNAM-1 (i.e., donor lymphocytes that persistently produce sDNAM-1 and recipient lymphocytes that had produced it before all-HSCT). On the other hand, the model 2 is consisted of three secretors of sDNAM-1 (i.e., donor lymphocytes that transiently release sDNAM-1 in addition to those in the model 1). Here, we used AIC, which can be used to compare two mathematical models [13].

## Results

### Mathematical model of sDNAM-1 dynamics

sDNAM-1 in sera of patients that received allo-HSCT can be derived from lymphocytes of either a donor, a recipient or both. Therefore, we assumed that sDNAM-1 in sera of patients consists of three types of sDNAM-1. The first and second types are transiently and persistently released from donor lymphocytes, respectively. The third one is a residual sDNAM-1 derived from recipient’s lymphocytes before allo-HSCT. Since it is difficult to discriminate the origins of serum sDNAM-1 detected by ELISA, we performed a computational simulation to estimate the concentration of each type of sDNAM-1. In our mathematical model, we denoted *x*_1_(*t*) as the concentration of the first type of sDNAM-1. We adopted a gamma distribution for *x*_1_(*t*), because the gamma function is a unimodal distribution and has a flexible shape, unlike the normal distribution. It can be used to express the feature of first type of sDNAM-1, namely transient growth. The second and third types of sDNAM-1 follows a logistic equation and an exponential decay pattern, which we denoted them as *x*_2_(*t*) *and x*_3_(*t*), respectively. The model is shown as follows:

$$\left\{ \begin{array}{l} \frac{dx_{1}(t)}{dt}=\lambda \frac{t^{k-1}e^{-\frac{t}{\theta }}}{\Gamma (k)\theta^{k}}-\mu x_{1}(t)\\ \frac{dx_{2}(t)}{dt}=(r-\mu )x_{2}(t)(1-\frac{x_{2}(t)}{N})\\ \frac{dx_{3}(t)}{dt}=-\mu x_{3}(t) \end{array}\right.$$ (1)

Definition of each parameter are given in **S1 Table** in the **Supplementary Methods**. *x*_1_(*t*)+*x*_2_(*t*)+*x*_3_(*t*) expresses the total amount of sDNAM-1. We fitted this model to the observed data and obtained the estimated parameters.

### Definition of *R*_*day_n*_ and comparison of *R*_*day_n*_ between patients’ group with or without aGVHD

We defined *R*_*day_n*_ using a mathematical expression as follows:

$$R_{day\_n}=\frac{100\int_{0}^{day\_n}x_{1}(t)\;dt}{\int_{0}^{day\_n}x_{1}(t)\;dt+\int_{0}^{day\_n}x_{2}(t)\;dt}$$ (2)

**Fig 2:**
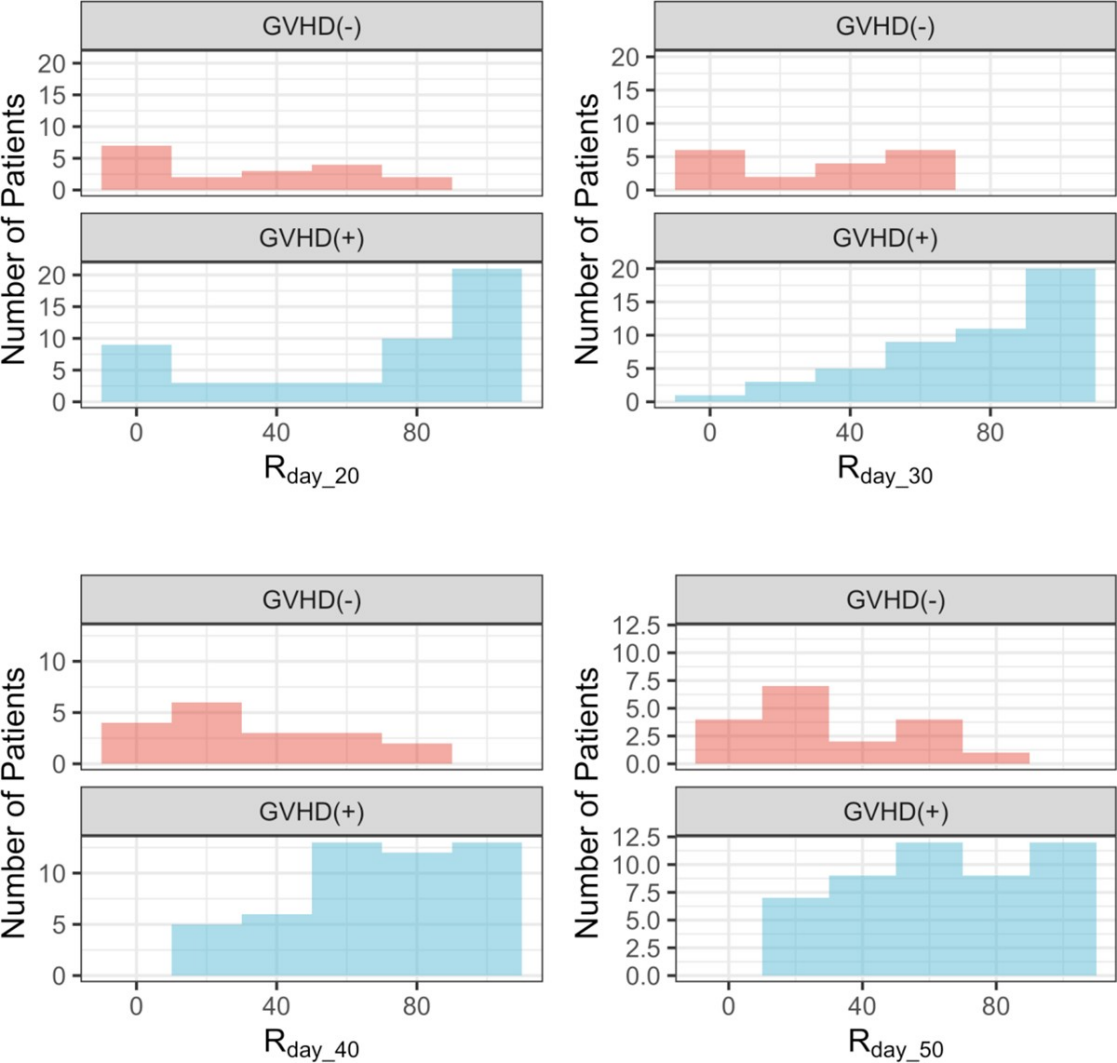
Distributions of *R*_*day_n*_ values for day n = 20, 30, 40, and 50. *R*_*day_n*_ does not follow a normal distribution. The vertical axis is the number of patients, and the horizontal axis is the value of *R*_*day_n*_.

**Fig 3:**
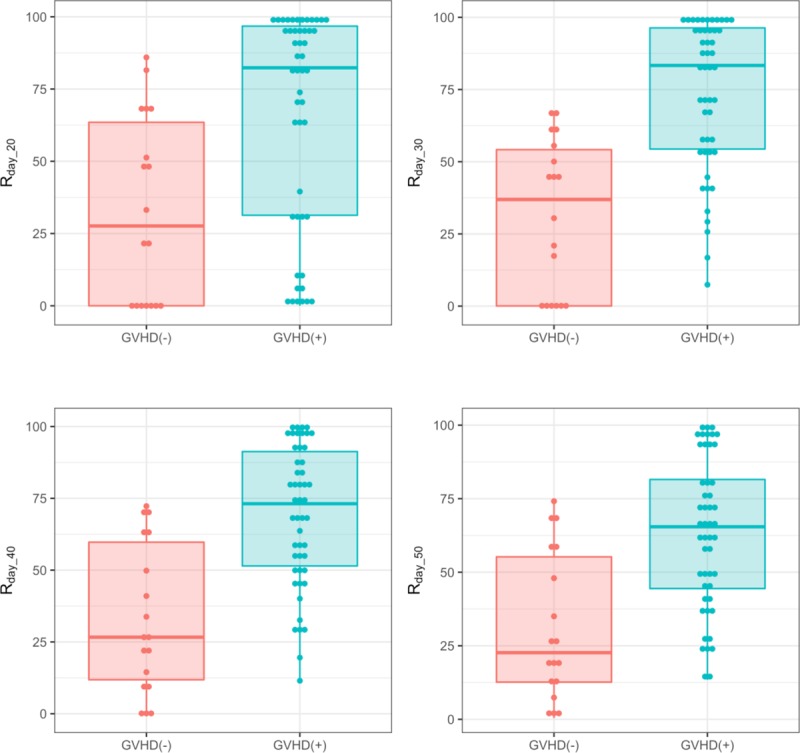
Box plots comparing *R*_day_n_ values between GVHD (+) and GVHD (–). GVHD (+) and GVHD (-) indicate patients with and without aGVHD. A thick line in each box indicates the median value of *R*_*day_*n_ (n = 20,30,40 and 50).

**Fig 4:**
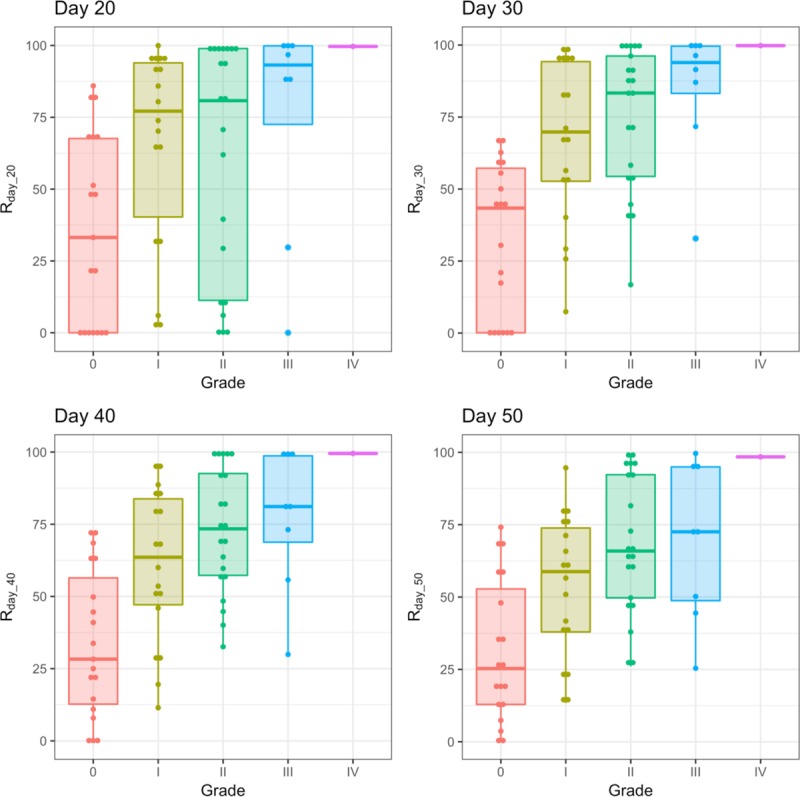
Box plots of *R*_*day_n*_ (n = 20, 30, 40, and 50) values per grade of aGVHD). *R*_*day_n*_ (%) values (*n* = 20, 30, 40, and 50) in patients with aGVHD without (grade 0) and with (grade I-IV) aGVHD. There is no box plot for GradeⅣ, as it contained only one patient.

Integration of *x*_1_(*t*) gives an area under the curve (AUC). The AUC of *x*_1_(*t*) from 0 to day_*n* is the amount of sDNAM-1 that is secreted by the donor lymphocytes during *n* days after allo-HSCT. In order to assess the effect of sDNAM-1 from donor lymphocytes on acute GVHD, we excluded the third type of sDNAM-1, which is derived from recipient lymphocytes. Since donor-derived allo-reactive T cells attack the recipient’s tissues during acute GVHD [14], it is reasonable to focus on the first and second type of sDNAM-1 derived from donor lymphocytes. Therefore, *R*_*day_n*_ is the proportion of sDNAM-1, which is released transiently from donor lymphocytes. We plotted each distribution of *R*_*day_n*_. The *R*_*day_n*_ distribution of patients with aGVHD (GVHD (+)) tended to shift to the right, whereas the distribution of patients without aGVHD (GVHD (-)) tended to shift to the left (**Fig 2**). We also compiled boxplots of *R*_*day_n*_ for GVHD (+) and GVHD (–) (*n* = 20, 30, 40, and 50). *R*_*day_n*_ of GVHD (+) was higher than that of GVHD (–) for each day (*n* = 20, 30, 40, and 50) (**Fig 3**). We performed *t*-test and Wilcoxon test and calculated the 95% CIs of mean & median of differences between GVHD (+) and GVHD (-) groups. The GVHD (+) group showed higher *R*_*day_20*_, *R*_*day_30*_, *R*_*day_40*_, and *R*_*day_50*_ than did the GVHD (–) group (**Table 2**). The differences in each estimated mean between the two groups exceeded 30% (**Table 2**). In addition, we analyzed the relationship between *R*_*day_n*_ (n = 20, 30, 40, and 50) with the grades of aGVHD. We found that the higher the grade, the more the value of *R*_*day_n*_ (n = 20, 30, 40, and 50) increased (**Fig 4**), suggesting that *R*_*day_n*_ reflected the grade of aGVHD. In addition, we also found the association of *R*_*day_n*_ with aGVHD in the skin and gastrointestinal tract, but not in the liver (**S2–S4 Figs**, **S2–S4 Tables**). In contrast, we did not observe the association of *R*_*day_n*_ with the factors of patient background (e.g., with or without CMV infection, total body irradiation, and HLA matching, and treatment for GVHD prophylaxis) (**S5–S8 Tables**).

**Table 2:** Values of *R*_*day_n*_ (n = 20, 30, 40, and 50 days).

	GVHD (–) (N = 19)	GVHD (+) (N = 48)	Difference in mean (95% CI)	*P*-value (*t*-test)	*P*-value (Wilcoxon-test)
*R*_*day_20*_	36% (± 33%)	66% (± 38%)	30% (10%–50%)	3.5 e–3	3.4e–4
*R*_*day_30*_	33% (± 27%)	74% (± 26%)	41% (27%–56%)	1.9e–7	1.6e–6
*R*_*day_40*_	34% (± 27%)	70% (± 25%)	36% (23%–50%)	1.1e–6	3.4e–6
*R*_*day_50*_	31% (± 25%)	64% (± 25%)	33% (19%–46%)	1.1e–5	2.3e–5

Estimated values and standard deviations of each *R*_*day_n*_ (n = 20, 30, 40, and 50) are shown. Estimated differences mean of *R*_*day_n*_ (n = 20, 30, 40, and 50) and these 95% confidence intervals are also shown. Results of statistical tests and *P*-values are also shown.

### Half-life of sDNAM-1

Based on our mathematical model, the half-life of sDNAM-1 was calculated as $\frac{log2}{\mu }$. The mean of half-life of sDNAM-1 was 12.5 +/- 9.2 days (n = 67). This calculation formula did not take the continuous production of sDNAM-1, which is variable in each patient, into account, resulting in the relatively high deviation of the half-life.

### Days elapsed from HSCT to the development of aGVHD

We plotted a histogram of the days elapsed from allo-HSCT to the onset of aGVHD. The mean number of days was 23 (**S1 Fig**). Twenty-four patients (50.0%) developed aGVHD within 20 days after allo-HSCT, 39 (81.3%) within 30 days, 41 (85.4%) within 40 days, and 48 (100%) within 50 days.

### Comparison between the models 1 and 2 using AIC

We compared 2 mathematical models to assess their goodness of fit to the data. The model 1 is consisted of two secretors of DNAM-1 (i.e., donor and recipient lymphocytes). On the other hand, the model 2 is consisted of three secretors of DNAM-1 (i.e., two types of donor lymphocytes that transiently and persistently release sDNAM-1 and recipient lymphocytes). The percentages of patients suitable for the models 1 and 2 is 29.9% and 70.1%, respectively (p-value = 0.0013) (**Table 3)**. These results suggest that the model 2 consisting of three types of sDNAM-1 explained the dynamics of the observed sDNAM-1 better than did the model 1 consisting of two types of sDNAM-1.

**Table 3:** Comparison of data fitting between two models using Akaike’s information criterion.

	Model with 2 types of sDNAM-1: no. of patients (%)	Model with 3 types of sDNAM-1: no. of patients (%)	*P*-value (binomial test)
GVHD (–)	7	12	–
GVHD (+)	13	35	–
Total	20 (29.9%) (19%-42%)	47 (70.1%) (58%-81%)	0.0013 (<0.05)

The model that included three types of sDNAM-1 explained the data of each patient more accurately (70.1%) than the model with only two types. The Percentages, 95% Confidence Intervals (CIs) and the result of statistical test are shown.

### Simulation of dynamics of sDNAM-1 based on *R*_*day_n*_

**Fig 5:**
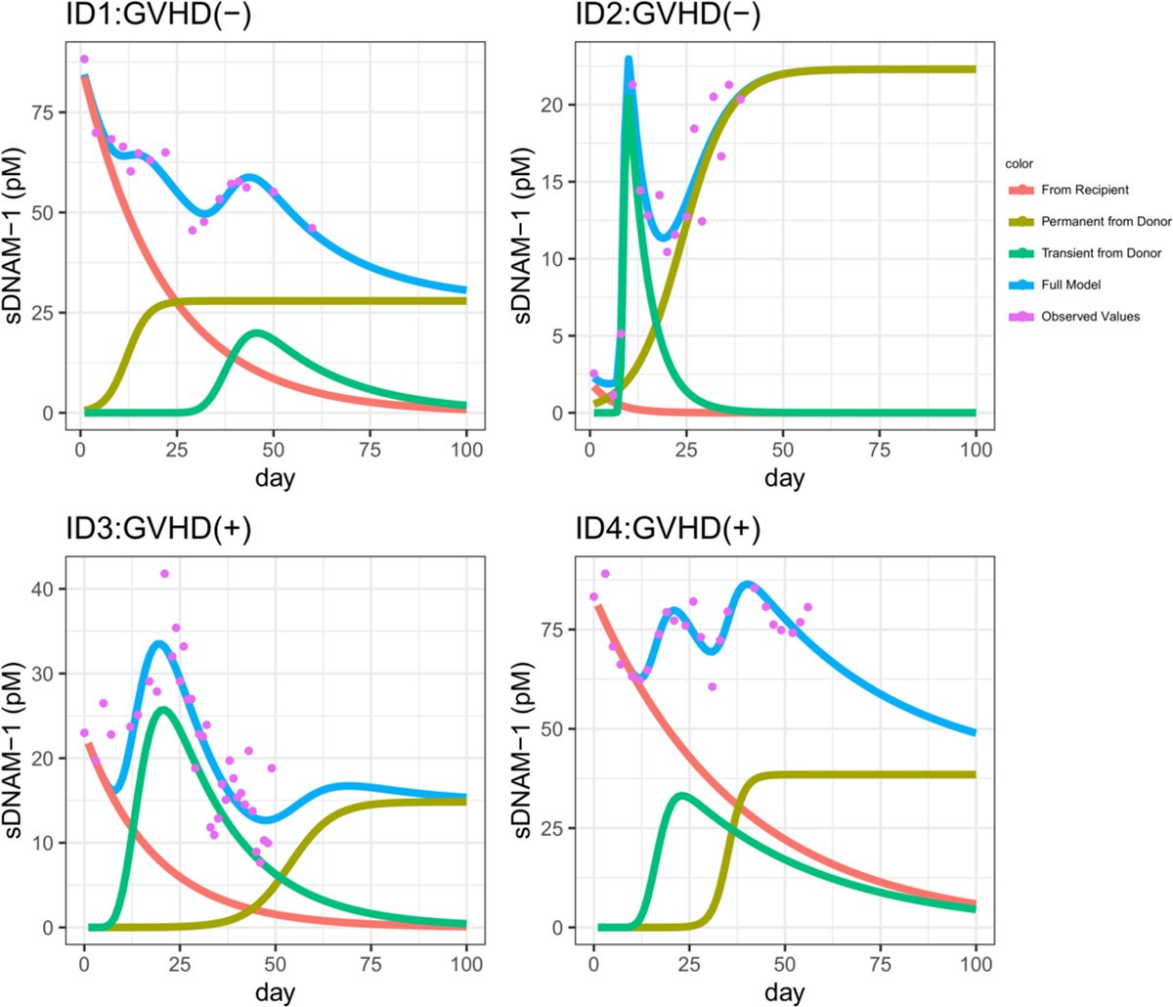
Observed data and simulation of the dynamics of the soluble form of DNAM-1 (sDNAM-1) from different sources. Representative patients Data of sDNAM-1 dynamics after transplantation are shown. The purple dots indicate the observed data. The blue line models the total amount of sDNAM-1; it represents the full model (= *x*_1_(*t*)+*x*_2_(*t*)+*x*_3_(*t*)). The green line models the first type of sDNAM-1, namely the transiently type (= *x*_1_(*t*)). The yellow-green line models the second type of sDNAM-1, namely the persistent type (= *x*_2_(*t*)). The red line models the third type of sDNAM-1, namely the residual type produced by recipient (= *x*_3_(*t*)). Thus, the blue line (total concentration of sDNAM-1) consists of the green, yellow-green, and red lines combined. Patients ID1 and ID2 did not develop aGVHD, whereas patients ID3 and ID4 did develop it.

We simulated the dynamics of sDNAM-1 using the patients’ data (**Fig 5**). For example, patient ID3 who developed aGVHD (GVHD (+)) had high percentages of *R*_*day_n*_ (*R*_*day_30*_ = 99.7%, *R*_*day_40*_ = 98.8% and *R*_*day_50*_ = 94.8%). Patient ID4 (GVHD (+)) also had high percentages of *R*_*day_n*_ (*R*_*day_30*_ = 98.7%, *R*_*day_40*_ = 78.3%, and *R*_*day_50*_ = 61.0%). In contrast, patient ID2 who did not develop aGVHD (GVHD (–)) had a low concentration of total sDNAM-1 (maximum = 21.3 pM) and low percentages of *R*_*day_n*_ (*R*_*day_30*_ = 44.3%, *R*_*day_40*_ = 28.3%, and *R*_*day_50*_ = 20.0%). Although the patient ID1 had a high concentration of total sDNAM-1 (maximum = 88.3 pM; see a highest purple dot), the patient did not develop aGVHD. Rather, the patient had low percentages of *R*_*day_n*_ (*R*_*day_30*_ = 0.211%, *R*_*day_40*_ = 7.91%, and *R*_*day_50*_ = 19.4%).

The AUC values of *R*_*day_20*,_ *R*_*day_30*,_ *R*_*day_40*,_ and *R*_*day_50*_ were 0.77, 0.86, 0.85, and 0.82, respectively (**Table 4**). We set the cutoffs of *R*_*day_n*_ (*n* = 20, 30, 40, and 50) of 70%, 66%, 51%, and 37%, respectively based on the method of “The closest-to-(0,1) criterion” [15]. The respective sensitivities and specificities of *R*_*day_20*,_ *R*_*day_30*,_ *R*_*day_40*,_ and *R*_*day_50*_ were 63%, 69%, 79%, and 85%, and 84%, 89%, 74%, and 68%, respectively. The accuracies were 69%, 75%, 78%, and 81%, respectively.

**Table 4:** Assessment of *R*_*day_n*_ by using receiver operating characteristics (ROCs).

	Method	AUC	Sensitivity	Specificity	Accuracy
*R*_*day_20*_	Mathematical model	0.77 (0.66–0.89)	63%	84%	69%
*R*_*day_30*_	Same as above	0.86 (0.77–0.94)	69%	89%	75%
*R*_*day_40*_	Same as above	0.85 (0.75–0.94)	79%	74%	78%
*R*_*day_50*_	Same as above	0.82 (0.71–0.93)	85%	68%	81%
Kanaya et al. [7]	Based on maximum value	0.68 (0.54–0.82)	69%	70%	69%
Paczensy et al. [16]	Logistic Regression	-	57%	75%	65%
Lee et al. [17]	super learner methods	*0.613–0.640	-	-	-

Comparison of areas under the curve (AUCs) between our *R*_*day_n*_ values and those of other studies. The definition of accuracy is TP+TN/(TP+TN+FP+FN). TP = number of true positives; TN = number of true negatives; FP = number of false positives; FN = number of false negatives. “–” means that no value was given in the article cited.

## Discussion

The biomarkers for aGVHD have been identified and validated as promising tools for diagnosis, assessment, prediction of response to therapy, and prognostic risk [7,18–20]. Several groups have also identified organ-specific biomarkers for aGVHD, such as elafin for skin aGVHD, regenerating islet-derived 3-alpha (Reg3α) for gastrointestinal tract aGVHD, and hepatocyte growth factor (HGF) and cytokeratin fragment 18 (KRT18) for liver aGVHD [7,20–22]. In addition, soluble suppression of tumorigenicity 2 (ST2) and the plasma microRNA signature were identified as predictive biomarkers for resistance to systemic steroid therapy to aGVHD and survival of allo-HSCT patients with aGVHD [20,23].

In the current study, we constructed a mathematical model to assess the dynamics of sDNAM-1 in the sera of patients after allo-HSCT and applied it to the data set of sDNAM-1, which had been previously analyzed [7]. For a mathematical model, we assumed that there were three types of sDNAM-1. The first and second types of sDNAM-1 are released from alloreactive and non-alloreactive donor lymphocytes, respectively. The third type is residual one released from recipient lymphocytes before allo-HSCT. Based on these three types of sDNAM-1, we constructed a mathematical model for the dynamics of sDNAM-1 using either these three or two (the second and the third) types of sDNAM-1 and found that the model using the three types efficiently fitted into the data better than the model using two types of sDNAM-1, demonstrating that the first type of sDNAM-1 is required for the construction of a better mathematical model. By using this model, we showed that the greater the proportion of the first type of sDNAM-1 to the total of the first and second types of sDNAM-1 during *n* days (*R*_*day_n*_) (*n* = 20, 30, 40, and 50) after allo-HSCT was, the more likely the patient developed aGVHD. *R*_*day_n*_ was a reliable index for high sensitivity of and specificity to the development of aGVHD. We also showed that *R*_*day_n*_ correlated well with the GVHD severity. Thus, *R*_*day_n*_ is a key biomarker for the development of aGVHD when sDNAM-1 was analyzed repeatedly over time after allo-HSCT.

Considering that allogeneic effector T cells derived from donors proliferate rapidly after transplantation and play a central role in the pathogenesis of aGVHD [24,25][26], donor lymphocytes producing the first type of sDNAM-1 are likely to be allogeneic effector T cells. On the other hand, donor lymphocytes that persistently release sDNAM-1 might be non-effector T cells. This scenario is in agreement with the previous report that certain T cells showed clonal expansions after allo-HSCT particularly in patients with aGVHD as demonstrated by the T cell antigen receptor repertory analyses [27].

Our study showed that our mathematical model for sDNAM-1 dynamics provides a useful biomarker for the development of aGVHD. However, a larger prospective study is required to generalize the significance of this mathematical model as an aGVHD biomarker. Nevertheless, our concept provides an important framework of a sensitive and specific biomarker for aGVHD.

## Supporting information

S1 Fig Histogram of onset day of aGVHD. (DOCX)

S2 Fig Relation between Skin GVHD and R_day_n_. (DOCX)

S3 Fig Relation between gastrointestinal GVHD and R_day_n_. (DOCX)

S4 Fig Relation between liver GVHD and R_day_n_. (DOCX)

S1 Table Definitions of parameters used in the mathematical model. (DOCX)

S2 Table Relation between skin GVHD and R_day_n_. (DOCX)

S3 Table Relation between gastrointestinal GVHD and R_day_n_. (DOCX)

S4 Table Relation between liver GVHD and R_day_n_. (DOCX)

S5 Table Relation between TBI and *R*_*day_n*_. (DOCX)

S6 Table Relation between CMV infection and *R*_*day_n*_. (DOCX)

S7 Table Relation between GVHD prophylaxis and *R*_*day_n*_. (DOCX)

S8 Table Relation between HLA matching and *R*_*day_n*_. (DOCX)
