# Supplementary material for: A mathematical model for dynamics of soluble form of DNAM-1 as a biomarker for graft-versus-host disease
Source: PLoS One. 2020 Feb 10;15(2):e0228508. doi: 10.1371/journal.pone.0228508 (PMC7010286; doi:10.1371/journal.pone.0228508)
Supplement: S1 Fig — (DOCX) [file pone.0228508.s001.docx]

**Supporting Information**

**S1 Figure. Histogram of onset day of aGVHD**

Time of onset of aGVHD after transplantation

The mean and median of the onset were day 23 and day 21, respectively and there are 49 patients (GVHD (+)).

**

**
