# Supplementary material for: A mathematical model for dynamics of soluble form of DNAM-1 as a biomarker for graft-versus-host disease
Source: PLoS One. 2020 Feb 10;15(2):e0228508. doi: 10.1371/journal.pone.0228508 (PMC7010286; doi:10.1371/journal.pone.0228508)
Supplement: S3 Fig — (DOCX) [file pone.0228508.s003.docx]

**S3 Figure. Relation between Gastrointestinal GVHD and R_day_n_**

GVHD (+) and GVHD (-) indicate patients with and without aGVHD. A thick line in each box indicates the median value of *R_day__*_n_ (n=20,30,40 and 50).
