# Supplementary material for: A mathematical model for dynamics of soluble form of DNAM-1 as a biomarker for graft-versus-host disease
Source: PLoS One. 2020 Feb 10;15(2):e0228508. doi: 10.1371/journal.pone.0228508 (PMC7010286; doi:10.1371/journal.pone.0228508)
Supplement: S1 Table — (DOCX) [file pone.0228508.s005.docx]

| Parameter | Description |
| --- | --- |
| $\lambda$ | Adjusted parameter of gamma distribution |
| *k* | Shape parameter of gamma distribution |
| *θ* | Scaling parameter of gamma distribution |
| *μ* | Rate of elimination of sDNAM-1 |
| *r* | Rate of production of sDNAM-1 |
| *N* | Estimated maximum concentration of second type of sDNAM-1 |
| $x_{2}\left( 0 \right)$ | Initial concentration of second type of sDNAM-1 |
| $x_{3}\left( 0 \right)$ | Initial concentration of third type of sDNAM-1 |

**S1 Table. Definitions of parameters used in the mathematical model**

In our model, we set the initial value of the first type of sDNAM-1 as zero, i.e. $x_{1}\left( 0 \right)=0$, because it was reasonable that there was little of this type present right after transplantation: We surmised that the first type of sDNAM-1 is produced by alloreactive T cells and that these cells do not react soon after transplantation (At least 0-3 days after transplantation, acute GVHD would not happen.), because the mean time of onset of aGVHD is 23 days (see **S1 Figure**). Since patients are given intensive chemotherapy before transplantation, their residual cell count and thus their concentration of sDNAM-1 is very low immediately after transplantation. We therefore assumed that at this time there were very few alloreactive T cells from the donor present and that little of the first type of sDNAM-1 was released. Therefore, we ignored the initial concentration of the first type of DNAM-1 and set $x_{1}\left( 0 \right)$ as zero.

In contrast, we set $x_{2}\left( 0 \right)+ x_{3}\left( 0 \right)$ as the “initial observed concentration.” We defined the “initial observed concentration” as below and applied these definitions to each patient from (i) to (iv), in order. If a patient satisfied several definitions, we adopted the definition with the smaller number among (i) to (iv).

(i) We used the sDNAM-1 concentration observed on day 1.

(ii) We used the sDNAM-1 concentration observed on day 0 (day of transplantation).

(iii) We used the sDNAM-1 concentrations observed between days 2 and 5 after transplantation, and we gave priority to the value taken closer to the day of transplantation.

(iv) We used the sDNAM-1 concentrations observed during days –7 to –1 (day 0 = day of transplantation), and we gave priority to the value taken closer to the day of transplantation.
