# Supplementary material for: A mathematical model for dynamics of soluble form of DNAM-1 as a biomarker for graft-versus-host disease
Source: PLoS One. 2020 Feb 10;15(2):e0228508. doi: 10.1371/journal.pone.0228508 (PMC7010286; doi:10.1371/journal.pone.0228508)
Supplement: S2 Table — (DOCX) [file pone.0228508.s006.docx]

|  | **Skin (–)**  (N = 22) | **Skin (+)**  (N = 45) | **Difference in mean**  **(95% CI)** | ***P*-value**  (*t*-test) |
| --- | --- | --- | --- | --- |
| *R_day_20_* | 38% (± 34%) | 67% (± 38%) | 29%  (10%–48%) | 0.0032 |
| *R_day_30_* | 37% (± 28%) | 75% (± 27%) | 37%  (23%–51%) | 1.4e-6 |
| *R_day_40_* | 37% (± 27%) | 70% (± 25%) | 33%  (20%–46%) | 3.7e-6 |
| *R_day_50_* | 34% (± 25%) | 65% (± 25%) | 31%  (17%–44%) | 1.7e-5 |

**S2 Table. Values of *R_day_n_* (n = 20, 30, 40, and 50 days) of Skin GVHD**

Estimated values and standard deviations of each *R_day_n_* (n = 20, 30, 40, and 50) are shown. Estimated differences mean of *R_day_n_* (n = 20, 30, 40, and 50) and these 95% confidence intervals are also shown. Results of statistical tests and *P*-values are also shown.
