# Supplementary material for: A mathematical model for dynamics of soluble form of DNAM-1 as a biomarker for graft-versus-host disease
Source: PLoS One. 2020 Feb 10;15(2):e0228508. doi: 10.1371/journal.pone.0228508 (PMC7010286; doi:10.1371/journal.pone.0228508)
Supplement: S3 Table — (DOCX) [file pone.0228508.s007.docx]

|  | **Gastrointestinal (–)**  (N = 56) | **Gastrointestinal (+)**  (N = 11) | **Difference in mean**  **(95% CI)** | ***P*-value**  (*t*-test) |
| --- | --- | --- | --- | --- |
| *R_day_20_* | 55% (± 39%) | 70% (± 39%) | 16%  (-9.9%–41%) | 0.23 |
| *R_day_30_* | 59% (± 32%) | 82% (± 23%) | 24%  (3.1%–44%) | 0.025 |
| *R_day_40_* | 56% (± 29%) | 78% (± 26%) | 22%  (3.5%–41%) | 0.021 |
| *R_day_50_* | 51% (± 28%) | 74% (± 29%) | 23%  (4.2%–41%) | 0.017 |

**S3 Table. Values of *R_day_n_* (n = 20, 30, 40, and 50 days) of Gastrointestinal GVHD**

Estimated values and standard deviations of each *R_day_n_* (n = 20, 30, 40, and 50) are shown. Estimated differences mean of *R_day_n_* (n = 20, 30, 40, and 50) and these 95% confidence intervals are also shown. Results of statistical tests and *P*-values are also shown.
