# Supplementary material for: A mathematical model for dynamics of soluble form of DNAM-1 as a biomarker for graft-versus-host disease
Source: PLoS One. 2020 Feb 10;15(2):e0228508. doi: 10.1371/journal.pone.0228508 (PMC7010286; doi:10.1371/journal.pone.0228508)
Supplement: S4 Table — (DOCX) [file pone.0228508.s008.docx]

|  | **Liver (–)**  (N = 56) | **Liver (+)**  (N = 11) | **Difference in mean**  **(95% CI)** | ***P*-value**  (*t*-test) |
| --- | --- | --- | --- | --- |
| *R_day_20_* | 58% (± 38%) | 53% (± 45%) | -5.7%  (-31%–20%) | 0.66 |
| *R_day_30_* | 60% (± 33%) | 74% (± 28%) | 14%  (-7.3%–35%) | 0.20 |
| *R_day_40_* | 57% (± 30%) | 72% (± 22%) | 19%  (-4.3%–34%) | 0.13 |
| *R_day_50_* | 52% (± 30%) | 66% (± 24%) | 14%  (-5.2%–33%) | 0.15 |

**S4 Table. Values of *R_day_n_* (n = 20, 30, 40, and 50 days) of Liver GVHD**

Estimated values and standard deviations of each *R_day_n_* (n = 20, 30, 40, and 50) are shown. Estimated differences mean of *R_day_n_* (n = 20, 30, 40, and 50) and these 95% confidence intervals are also shown. Results of statistical tests and *P*-values are also shown.
