# Supplementary material for: A mathematical model for dynamics of soluble form of DNAM-1 as a biomarker for graft-versus-host disease
Source: PLoS One. 2020 Feb 10;15(2):e0228508. doi: 10.1371/journal.pone.0228508 (PMC7010286; doi:10.1371/journal.pone.0228508)
Supplement: S5 Table — (DOCX) [file pone.0228508.s009.docx]

|  | **CMV (–)**  (N = 39) | **CMV (+)**  (N = 28) | **Difference in mean**  **(95% CI)** | ***P*-value**  (*t*-test) |
| --- | --- | --- | --- | --- |
| *R_day_20_* | 54% (± 37%) | 56% (± 42%) | 2.6%  (-12%–17%) | 0.72 |
| *R_day_30_* | 63% (± 30%) | 62% (± 35%) | -1.5%  (-17%–15%) | 0.85 |
| *R_day_40_* | 59% (± 27%) | 60% (± 33%) | 0.79%  (-14%–16%) | 0.92 |
| *R_day_50_* | 53% (± 27%) | 56% (± 32%) | 2.6%  (-12%–17%) | 0.72 |

**S5 Table. Values of *R_day_n_* (n = 20, 30, 40, and 50 days) of CMV infection**

Estimated values and standard deviations of each *R_day_n_* (n = 20, 30, 40, and 50) are shown. Estimated differences mean of *R_day_n_* (n = 20, 30, 40, and 50) and these 95% confidence intervals are also shown. Results of statistical tests and *P*-values are also shown. CMV means cytomegalovirus infection.
