# Supplementary material for: A mathematical model for dynamics of soluble form of DNAM-1 as a biomarker for graft-versus-host disease
Source: PLoS One. 2020 Feb 10;15(2):e0228508. doi: 10.1371/journal.pone.0228508 (PMC7010286; doi:10.1371/journal.pone.0228508)
Supplement: S6 Table — (DOCX) [file pone.0228508.s010.docx]

|  | **TBI (–)**  (N = 28) | **TBI (+)**  (N = 39) | **Difference in mean**  **(95% CI)** | ***P*-value**  (*t*-test) |
| --- | --- | --- | --- | --- |
| *R_day_20_* | 66% (± 36%) | 45% (± 40%) | 22%  (3.2%–40%) | 0.022 |
| *R_day_30_* | 68% (± 29%) | 55% (± 36%) | 12%  (-3.3%–28%) | 0.12 |
| *R_day_40_* | 63% (± 29%) | 55% (± 31%) | 8.0%  (-6.6%–23%) | 0.28 |
| *R_day_50_* | 58% (± 29%) | 50% (± 30%) | 7.2%  (-7.1%–22%) | 0.32 |

**S6 Table. Relation between TBI & *R_day_n_* (n = 20, 30, 40, and 50 days)**

Estimated values and standard deviations of each *R_day_n_* (n = 20, 30, 40, and 50) are shown. Estimated differences mean of *R_day_n_* (n = 20, 30, 40, and 50) and these 95% confidence intervals are also shown. Results of statistical tests and *P*-values are also shown. TBI means total body irradiation.
