# Supplementary material for: A mathematical model for dynamics of soluble form of DNAM-1 as a biomarker for graft-versus-host disease
Source: PLoS One. 2020 Feb 10;15(2):e0228508. doi: 10.1371/journal.pone.0228508 (PMC7010286; doi:10.1371/journal.pone.0228508)
Supplement: S7 Table — (DOCX) [file pone.0228508.s011.docx]

|  | **Tacrolimus**  (N = 43) | **CsA**  (N = 24) | **Difference in mean**  **(95% CI)** | ***P*-value**  (*t*-test) |
| --- | --- | --- | --- | --- |
| *R_day_20_* | 62% (± 37%) | 48% (± 41%) | 14%  (-6.0%–33%) | 0.17 |
| *R_day_30_* | 66% (± 31%) | 56% (± 34%) | 9.4%  (-6.9%–26%) | 0.25 |
| *R_day_40_* | 62% (± 29%) | 54% (± 31%) | 7.8%  (-7.2%–23%) | 0.30 |
| *R_day_50_* | 57% (± 29%) | 50% (± 29%) | 7.2%  (-7.6%–22%) | 0.34 |

**S7 Table. Relation between GVHD prophylaxis & *R_day_n_* (n = 20, 30, 40, and 50 days)**

Estimated values and standard deviations of each *R_day_n_* (n = 20, 30, 40, and 50) are shown. Estimated differences mean of *R_day_n_* (n = 20, 30, 40, and 50) and these 95% confidence intervals are also shown. Results of statistical tests and *P*-values are also shown. CsA means cyclosporine A.
