# Supplementary material for: A mathematical model for dynamics of soluble form of DNAM-1 as a biomarker for graft-versus-host disease
Source: PLoS One. 2020 Feb 10;15(2):e0228508. doi: 10.1371/journal.pone.0228508 (PMC7010286; doi:10.1371/journal.pone.0228508)
Supplement: S8 Table — (DOCX) [file pone.0228508.s012.docx]

|  | **HLA Mismatch**  (N = 23) | **HLA Full match**  (N = 44) | **Difference in mean**  **(95% CI)** | ***P*-value**  (*t*-test) |
| --- | --- | --- | --- | --- |
| *R_day_20_* | 63% (± 40%) | 54% (± 38%) | 9.3%  (-11%–29%) | 0.35 |
| *R_day_30_* | 65% (± 32%) | 61% (± 33%) | 3.5%  (-13%–20%) | 0.67 |
| *R_day_40_* | 61% (± 28%) | 59% (± 31%) | 1.8%  (-14%–17%) | 0.82 |
| *R_day_50_* | 55% (± 28%) | 55% (± 30%) | 0.32%  (-15%–15%) | 0.97 |

**S8 Table. Relation between HLA & *R_day_n_* (n = 20, 30, 40, and 50 days)**

Estimated values and standard deviations of each *R_day_n_* (n = 20, 30, 40, and 50) are shown. Estimated differences mean of *R_day_n_* (n = 20, 30, 40, and 50) and these 95% confidence intervals are also shown. Results of statistical tests and *P*-values are also shown.
